# Protein lactylation in cancer: mechanisms and potential therapeutic implications

**DOI:** 10.1038/s12276-025-01410-7

**Published:** 2025-03-24

**Authors:** Hyunsoo Rho, Nissim Hay

**Affiliations:** 1 Department of Biochemistry and Molecular Genetics, College of Medicine, University of Illinois at Chicago, Chicago, IL USA; 2 Research and Development Section, Jesse Brown VA Medical Center, Chicago, IL USA; 3 Present Address: College of Pharmacy, Graduate School of Pharmaceutical Sciences, Ewha Womans University, Seoul, Republic of Korea

**Keywords:** Cancer therapy, Oncogenesis

## Abstract

Increased glycolysis, which leads to high lactate production, is a common feature of cancer cells. Recent evidence suggests that lactate plays a role in the post-translational modification of histone and nonhistone proteins via lactylation. In contrast to genetic mutations, lactylation in cancer cells is reversible. Thus, reversing lactylation can be exploited as a pharmacological intervention for various cancers. Here we discuss recent advances in histone and nonhistone lactylation in cancer, including l-, d- and s-lactylation, as well as alanyl-tRNA synthetase as a novel lactyltransferase. We also discuss potential approaches for targeting lactylation as a therapeutic opportunity in cancer treatment.

## Lactate induces reversible modifications in cancer proteins

In the 1920s, Otto Warburg discovered that cancer cells use a lot of sugar even when oxygen is present. This process, called aerobic glycolysis, produces a substance called lactate. Scientists used to think lactate was just waste, but recent studies show it has important roles in cancer. Researchers found that lactate can change proteins in cells through a process called lactylation, which affects how genes are turned on or off. In this study, the authors explored how lactylation impacts cancer. They used various methods to study proteins and genes in cancer cells. They found that lactylation can change both histone proteins and nonhistone proteins, affecting gene expression and protein stability. This process can help cancer cells grow and resist treatment. The researchers concluded that targeting lactylation could be a new way to treat cancer.

This summary was initially drafted using artificial intelligence, then revised and fact-checked by the author.

## Introduction

**Fig. 1:**
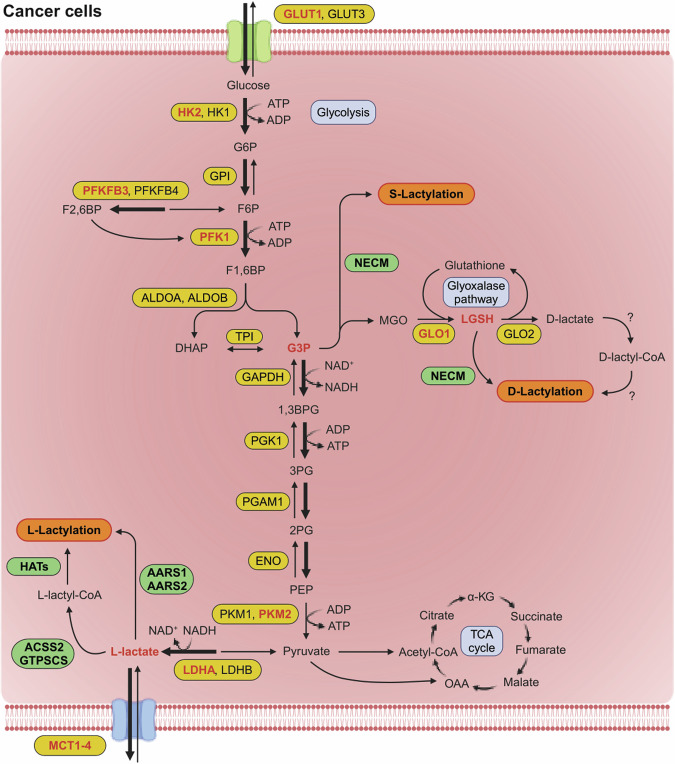
Reprogrammed glucose metabolism and l-, d- and s-lactylation. Compared with normal cells, cancer cells accelerate glycolysis by inducing different isozymes of glycolytic enzymes. Lactate, the end product of glycolysis, causes l-lactylation on lysine residues of target proteins by HATs or AARS. G3P or DHAP, the intermediate metabolites of glycolysis, upregulate LGSH, which causes d-lactylation on lysine residues of target proteins by NECMs. In addition, G3P causes s-lactylation on cysteine residues of target proteins by NECMs. Enzymes that catalyze metabolic reactions are depicted inside yellow ovals. Enzymes that are frequently induced in cancer cells are written in red inside yellow ovals. The precursor metabolites for lactylation are written in red. The enzymes that catalyze lactylation are depicted in green ovals. The thickness of the arrows represents the relative flux. 2PG, 2-phosphoglycerate; 3PG, 3-phosphoglycerate; α-KG, α-ketoglutarate; ALDO, aldolase; ENO, enolase; F1,6BP, fructose-1,6-bisphosphate; F2,6BP, fructose-2,6-bisphosphate; F6P, fructose-6-phosphate; G6P, glucose-6-phosphate; GAPDH, glyceraldehyde-3-phosphate dehydrogenase; GLUT, glucose transporter; GPI, glucose-6-phosphate isomerase; HK, hexokinase; MCT, monocarboxylate transporter; OAA, oxaloacetate; PEP, phosphoenolpyruvate; PFKFB, 6-phosphofructo 2-kinase/fructose-2,6-bisphosphatase; PGAM1, phosphoglycerate mutase 1; PGK1, phosphoglycerate kinase 1; PK, pyruvate kinase; TCA, tricarboxylic acid; TPI, triose phosphate isomerase.

In the 1920s, Otto Warburg discovered that glycolysis is increased in cancer cells even in the presence of oxygen^1,2^. This distinct metabolic feature is attributed to the induction of specific isozyme expression of glycolytic enzymes such as hexokinase 2 (HK2), phosphofructokinase 1 (PFK1), 6-phosphofructo-2-kinase/fructose-2,6-bisphosphatase 3 (PFKFB3), pyruvate kinase M2 (PKM2) and lactate dehydrogenase A (LDHA) in cancer cells compared with their counterparts in normal cells^3^. Increased glycolysis caused by altered glycolytic enzyme expression inevitably results in the production of a large amount of lactate, which has historically been considered metabolic waste (Fig. 1)^4^. However, emerging evidence suggests that lactate can act as a ligand for G protein-coupled receptor 81 (ref. ^5^), a signaling molecule that directly binds to soluble adenylyl cyclase^6^, and as an energy source that fuels mitochondria^7^. Recent studies have also shown that lactate exerts a post-translational modification (PTM), known as lactylation^8^.

In cancer cells, altered metabolic pathways are frequently linked to epigenetic modifications that control the chromatin structure and accessibility of the transcriptional machinery, thereby inducing aberrant gene expression^9,10^. Like previously identified types of histone acylation, including acetylation, propionylation, butyrylation and crotonylation, lactate-induced lactylation is capable of modifying the lysine residue in the N-terminal tail of core histones^8,11^. As mammalian cells express l-lactate dehydrogenase (LDH) in the last step of glycolysis^12^, the l-form of lactate is produced and activated as l-lactyl-CoA, which causes l-lactylation by histone acetyltransferases (HATs)^8^. HATs and histone deacetylases (HDACs) refer to the enzymes ‘writers’ and ‘erasers’ that add or remove modifications, respectively^11^. These enzymes are also involved in lysine lactylation (Kla) of nonhistone proteins^13^, which influences protein function and stability^14,15^. In addition, recent studies suggest that alanyl-tRNA synthetase (AARS) is a novel Kla writer that activates lactate into lactate-AMP, which is subsequently transferred to lysine residues of target proteins (Fig. 1)^16–18^.

Although mammalian cells mainly produce l-lactate via glycolysis, they can produce d-lactate via the glyoxalase (GLO) pathway, which is a branched metabolic pathway involving the glycolytic intermediates glyceraldehyde-3-phosphate (G3P) and dihydroxyacetone phosphate (DHAP)^19,20^. Whether d-lactate from the GLO pathway can modify lysine residues of target proteins via d-lactylation (also referred to as d-lactoylation), analogous to l-lactate and l-lactylation, remains elusive. However, recent evidence suggests that d-lactylation occurs via nonenzymatic covalent modifications (NECMs) from s-d-lactoylglutathione (LGSH), which is another metabolite generated via the GLO pathway (Fig. 1)^21^. In addition to l- and d-lactylation on the lysine residues of histone and nonhistone protein, a recent observation identified that lactylation also occurs on the cysteine residue of the target protein, known as s-lactylation (also referred to as *s*-lactoylation), which can also be installed nonenzymatically by G3P (Fig. 1)^22^. In this review, we summarize recent findings concerning histone and nonhistone lactylation in cancer. We also highlight potential therapeutic strategies targeting lactylation as a cancer therapy.

## Histone lysine lactylation in cancer

**Fig. 2:**
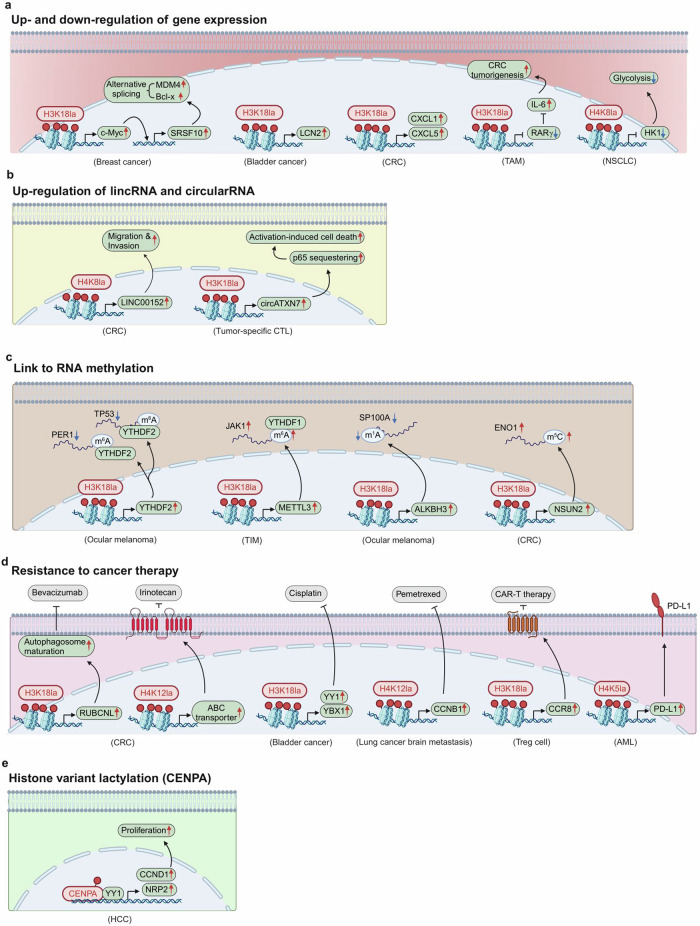
Histone lysine lactylation in cancer. **a**–**d** Histone lysine lactylation is related to protumorigenic gene expression (**a**), lincRNA and circular RNA expression (**b**) RNA methylation (**c**) and resistance to cancer therapy (**d**). **e** The histone H3 variant CENPA is lactylated to induce HCC proliferation. ABC transporter, ATP-binding cassette transporter; ALKBH3, α-ketoglutarate-dependent dioxygenase AlkB homolog 3; CCN, cyclin; circATXN7, circular RNA ataxin 7; HK1, hexokinase 1; JAK1, janus kinase 1; MDM4, murine double minute 4; METTL3, methyltransferase-like protein 3; NSUN2, NOP2/Sun RNA methyltransferase 2; PER1, period circadian clock 1; SP100A, SP100 nuclear antigen; TP53, tumor protein 53; YTHDF, YTH *N*^6^-methyladenosine RNA-binding protein; YY1, ying yang 1.

In 2019, Zhao and colleagues reported a novel epigenetic modification known as histone Kla by high-performance liquid chromatography–tandem mass spectrometry, with a mass shift of 72.021 Da at lysine residues of tryptically digested core histones^8^. In 2023, they found additional histone Kla sites by developing a new strategy called ‘Comprehensive Histone Mark Analysis’^23^. In 2024, they further demonstrated that histone Kla is dominantly mediated by l-lactylation in response to enhanced glycolysis^24^ and that guanosine triphosphate (GTP)-specific succinyl-CoA synthetase (GTPSCS) generates lactyl-CoA, the substrate of l-lactylation from l-lactate (Fig. 1)^25^. The biological function of histone Kla was initially established in macrophages that undergo increased glycolysis during M1 polarization^26^. Interestingly, lactate accumulation in the late phase of M1 polarization is correlated with increased histone Kla levels and the expression of M2-like genes, such as wound healing-related genes and *Arg1* (ref. ^27^). Conversely, the level of histone lysine acetylation (Kac), which marks the promoters of proinflammatory genes in the early M1 phase, is inversely correlated with the induction of histone Kla^8^. The discovery of histone Kla at the promoters of many genes that lack histone Kac implies that this new histone modification is involved in the expression of a distinct set of genes, particularly in cancer cells, where increased glycolysis results in the accumulation of lactate (Fig. 2a–e). The diverse functions of lactate-induced histone Kla in cancer are described in detail below.

### Histone Kla and the up- and downregulation of gene expression

Histone Kla is upregulated and associated with tumorigenic gene expression in many cancer cells. Among the various lysine residues involved in histone lactylation, histone H3 lysine 18 lactylation (H3K18la) is probably the most well-known histone Kla. Liver fibrosis, which can induce a predisposition to metabolic dysfunction-associated steatohepatitis-induced hepatocellular carcinoma (HCC), was recently shown to be dependent on histone lactylation, particularly H3K18la^28^. In breast cancer cells, H3K18la is enriched at the promoter of the oncogenic transcription factor c-Myc to promote its expression. C-Myc, in turn, elevates the expression of serine/arginine-rich splicing factor 10 (SRSF10). SRSF10 subsequently mediates the alternative splicing of MDM4 and Bcl-x (Fig. 2a)^29^. Bladder cancer progression is reportedly dependent on lipocalin-2 (LCN2), which is induced by H3K18la at its promoter (Fig. 2a)^30^. In colorectal cancer (CRC), H3K18la is induced not only in cancer cells but also in tumor-associated macrophages (TAMs). H3K18la in CRCs upregulates chemokine (C–X–C motif) ligand 1 (CXCL1) and CXCL5, which promote CRC metastasis (Fig. 2a)^31^. H3K18la at the promoter of retinoic acid receptor-γ (RARγ) is enriched in TAMs by taking up lactate from CRCs. Interestingly, increased H3K18la in TAMs suppresses RARγ expression, and this inhibition activates oncogenic signal transducer and activator of transcription 3 (STAT3) signaling in CRCs (Fig. 2a)^32^. Another study also proposed the possibility of transcriptional repression of histone Kla. In non-small-cell lung cancer (NSCLC), the glycolytic enzyme HK1 is repressed when H4K8la is induced (Fig. 2a)^33^. Although the net effect of histone Kla induction promotes cancer progression, the reason its gene regulatory function appears opposing remains to be elucidated.

### Histone Kla and noncoding RNAs and modification of RNA methylation

Histone Kla regulates not only protein-coding genes but also long intergenic noncoding RNAs (lincRNAs). In CRC, bacteria-derived lipopolysaccharide (LPS) activates oncogenic LINC00152 transcription by H4K8la but not H3K18la or H4K5la (Fig. 2b)^34^. In addition, a recent study shed light on the transcriptional activity of histone Kla in a new class of RNAs, circular RNAs (circRNAs). Lactate produced by oncogenic Kras in CRC elevates H3K18la, which in turn activates circATXN7 transcription in tumor-specific cytotoxic T lymphocytes (CTLs). CircATXN7 subsequently interacts with NF-κB p65 subunits and sequesters p65 in the cytosol, which sensitizes tumor-specific CTLs to activation-induced cell death (Fig. 2b)^35^. Thus, the activation-induced cell death of tumor-specific CTLs promotes tumor immune evasion and resistance to immunotherapy.

Multiple studies have demonstrated that histone Kla-induced genes are closely linked to RNA methylation, including *N*^6^-methyladenosine (m^6^A), *N*^1^-methyladenosine (m^1^A) and 5-methylcytosine (m^5^C) modifications. In ocular melanoma, histone Kla induces the expression of YTH *N*^6^-methyladenosine RNA-binding protein F2 (YTHDF2), which binds to the m^6^A-modified mRNAs of Period 1 (*PER1*) and tumor protein P53 (*TP53*) to promote their degradation, thereby leading to oncogenesis (Fig. 2c)^36^. The relationship between histone Kla and tumor progression via m^6^A modification has also been reported in tumor-infiltrating myeloid (TIM) cells. In the TIM, H3K18la upregulates the expression of the methyltransferase-like 3 (METTL3) gene, which installs m^6^A on *Jak1* mRNA, which is then bound to YTHDF1. Subsequently, increasing the translational efficiency of the JAK1 protein induces the immunosuppressive functions of TIMs (Fig. 2c)^37^. In addition to m^6^A RNA modification, H3K18la is involved in m^1^A RNA modification in ocular melanoma. H3K18la is enriched in the promoter of AlkB homolog 3 (ALKBH3), which has the ability to demethylate m^1^A. As a consequence of ALKBH3 induction, m^1^A methylation of the nuclear antigen SP100A mRNA is diminished, which in turn reduces the translational efficiency of SP100A. SP100A is a component of promyelocytic leukemia bodies, and its reduced production interferes with the assembly of promyelocytic leukemia bodies, consequently promoting tumorigenesis in ocular melanoma (Fig. 2c)^38^. In CRC, H3K18la is involved in the induction of m^5^C methyltransferase NSUN2 expression, which increases the stability of enolase1 (*ENO1*) mRNA by m^5^C modification. Increased *ENO1* expression subsequently elicits a positive feedback loop involving glycolysis and histone lactylation in CRC, which is correlated with poor patient prognosis (Fig. 2c)^39^.

### Histone Kla and resistance to cancer therapy

Histone Kla induces genes that are involved in resistance to cancer therapy. Bevacizumab, an antivascular endothelial growth factor drug, is used in first- and second-line treatment regimens for metastatic CRC^40^. However, prolonged use of this anti-angiogenic therapy eventually results in resistance to the therapy and thus limits its efficacy^41^. Upon bevacizumab administration, hypoxic cancer cells may increase glycolysis, which leads to increased H3K18la levels and the transcriptional upregulation of Rubicon-like autophagy enhancer (RUBCNL), thereby enhancing autophagosome maturation by binding to beclin-1 (Fig. 2d)^42^. Consequently, CRC receiving bevacizumab acquires cancer cell survival and therapy resistance via autophagy. H3K18la also contributes to cisplatin resistance by inducing transcription factor Y-box binding protein 1 (YBX1) and YY1 in bladder cancer^43^, and H4K12la increases ATP-binding cassette (ABC) transporter expression, rendering CRC resistant to chemotherapy (Fig. 2d)^44^. In a subpopulation of cells involved in the brain metastasis of lung cancer, aldo-keto reductase family 1 member B10 (AKR1B10) is overexpressed and induces H4K12la-dependent cyclin B1 (*CCNB1*) transcription by promoting LDHA expression, leading to resistance to pemetrexed chemotherapy agents (Fig. 2d)^45^. In the tumor microenvironment of glioblastoma (GBM), C–C motif chemokine receptor 8 (CCR8) expression mediated by H3K18la in regulatory T (Treg) cells contributes to the immunosuppressive tumor microenvironment to impede chimeric antigen receptor-T (CAR-T) cell immunotherapy (Fig. 2d)^46^. In acute myeloid leukemia (AML), STAT5 promotes glycolysis and lactate accumulation, which increases H4K5la at the promoter region of programmed death-ligand 1 (PD-L1), leading to the escape from immune surveillance (Fig. 2d)^47^.

### Histone variant Kla and tumorigenesis

In addition to Kla modification of canonical histones, the histone H3 variant called centromere protein A (CENPA) is also lactylated at K124, increasing its ability to interact with YY1 and thus induce cyclin D1 and neuropilin 2 (NRP2) in HCC (Fig. 2e)^48^.

## Nonhistone lysine lactylation in cancer

**Fig. 3:**
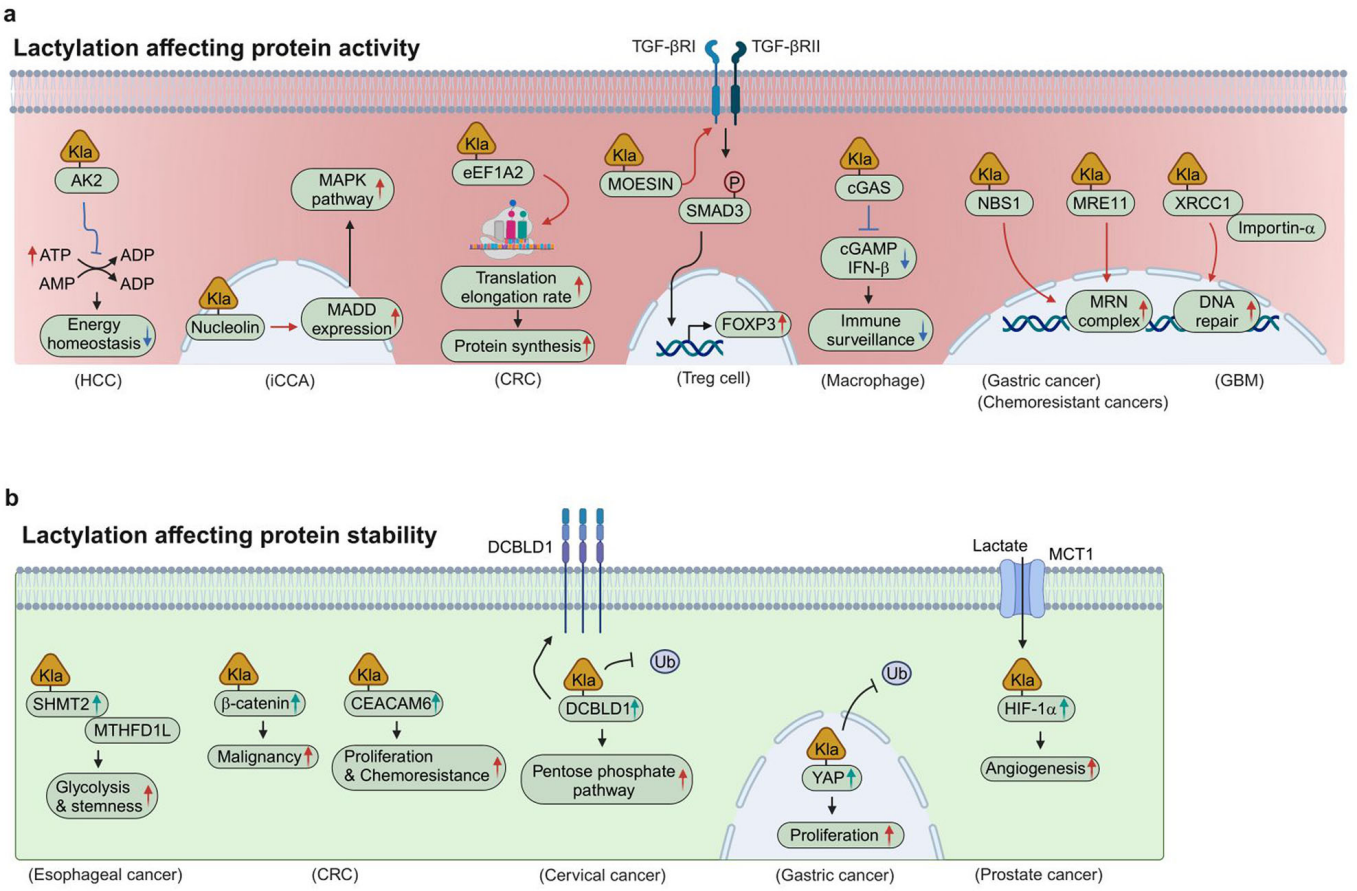
Nonhistone lysine lactylation in cancer. **a**, **b** Nonhistone lysine lactylation affects protein activity (**a**) and stability (**b**). eEF1A2, eukaryotic translation elongation factor 1 alpha 2; Ub, ubiquitin.

After histone Kla was identified, mounting evidence also suggests that nonhistone proteins are targets of Kla modification^15^ and play crucial roles in tumor progression and poor prognosis by affecting target protein activity and stability (Fig. 3a, b). The biological functions of nonhistone Kla in cancer are described in detail below.

### Nonhistone Kla and protein activity

Multiomics analysis conducted in HCC identified 9,256 Kla sites on nonhistone proteins out of a total of 9,275 Kla sites, indicating that Kla modification prevails in nonhistone proteins in liver cancer^15^. In this study, adenylate kinase 2 (AK2), which transfers the phosphate group among adenine nucleotides to generate ADP, was hyperlactylated at K28 (ref. ^15^). This Kla modification subsequently inhibits AK2 activity, leading to poor prognosis caused by energy metabolism disturbance (Fig. 3a). In intrahepatic cholangiocarcinoma (iCCA), proteomic analysis revealed that nucleolin is lactylated at K477, which is required for its ability to upregulate MAP kinase-activating death domain (MADD) expression. Thus, high MADD expression as a consequence of nucleolin lactylation activates the canonical mitogen-activated protein kinase (MAPK) pathway in iCCA (Fig. 3a)^49^. In CRC, the eukaryotic translation elongation factor eEF1A2 is hyperlactylated at K408, thereby increasing translation elongation rates to meet the increased biosynthetic demand (Fig. 3a)^50^. Nonhistone Kla affects protein activity in Treg cells to promote the immunosuppressive tumor microenvironment. Mechanistically, lactate modifies the membrane-organizing extension spike protein (MOESIN) by lactylation at K72, which enhances the interaction with tumor growth factor-β receptor 1 (TGF-βRI) and increases TGF-β signaling^51^. Subsequently, SMAD3 is phosphorylated and translocated to the nucleus to increase forkhead box P3 (FOXP3) expression, which maintains the immunosuppressive function of Treg cells (Fig. 3a). Additionally, nonhistone Kla is reported to promote the immunosuppressive function of macrophages in oncogenic viral infection^18^. Cyclic GMP–AMP synthase (cGAS) is a DNA sensor that produces cyclic GMP–AMP (cGAMP), which triggers interferon-β (IFN-β) production^52^. When cGAS is lactylated at K131, it decreases the electrostatic affinity of cGAS for DNA, which in turn reduces cGAMP and IFN-β production (Fig. 3a)^18^. Therefore, lactylated cGAS reduces innate immune surveillance. Recently, multiple research groups have reported that nonhistone Kla is involved in modulating the DNA repair system of cancer cells to resist therapeutic interventions. In GBM, X-ray repair cross-complementing protein 1 (XRCC1) is lactylated at K247, which increases its affinity for importin-α (Fig. 3a)^53^. Enhanced interaction leads to the nuclear translocation of XRCC1, which in turn promotes DNA repair against chemoradiotherapy for GBM^53^. The meiotic recombination 11 (MRE11)‒RAD50‒Nijmegen breakage syndrome 1 (NBS1) complex (MRN complex) plays a key role in orchestrating the DNA repair system^54^. In gastric cancer, hyperlactylation at K388 of NBS1 promotes the formation of the MRN complex, facilitating DNA repair in response to cisplatin treatment or ionizing radiation therapy (Fig. 3a)^55^. In addition, lactylation at K673 of MRE11 is increased in chemoresistant cancers and promotes DNA repair by enhancing the DNA binding ability of MRE11 (Fig. 3a)^56^.

### Nonhistone Kla and protein stability

Emerging evidence shows that nonhistone Kla affects protein stability in cancers under hypoxia. In esophageal cancer, hypoxia stabilizes serine hydroxymethyltransferase 2 (SHMT2) via lactylation, which enhances its interaction with the key enzyme in one-carbon metabolism, methylenetetrahydrofolate dehydrogenase (NADP^+^ dependent) 1-like (MTHFD1L), thereby facilitating a malignant phenotype (Fig. 3b)^57^. Hypoxia also increases β-catenin stability by Kla modification, which regulates proliferation and cell stemness in CRC (Fig. 3b)^58^. Although the mechanism by which Kla stabilizes its target protein needs further investigation, a recent study demonstrated that the lactylation of discoidin-CUB and LCCL domain-containing 1 (DCBLD1) at K172 enhances its stability by inhibiting ubiquitination in cervical cancer (Fig. 3b)^14^. Another study also demonstrated that Yes-associated protein (YAP) is lactylated at K90, which in turn increases the stability and nuclear translocation of YAP in gastric cancer (Fig. 3b)^16^. This lactylation is mediated by AARS1, which is elevated in gastric cancer and is associated with poor prognosis.

Kla-mediated protein stability is also observed when lactate is derived from neighboring cells. In CRC, high ALDOB-expressing cells activate pyruvate dehydrogenase kinase 1 (PDK1), which inhibits pyruvate oxidation but increases lactate production^59^. Lactate from ALDOB-expressing CRC cells enters adjacent neighboring cells and enhances CEA cell adhesion molecule 6 (CEACAM6) stability via lactylation, leading to CRC proliferation and chemoresistance (Fig. 3b)^59^. In prostate cancer, lactate enters cancer cells through monocarboxylate transporter 1 (MCT1) and stabilizes hypoxia inducible factor 1 α (HIF1α) via lactylation, thereby inducing the HIF1α pathway to stimulate angiogenesis (Fig. 3b)^60^.

## Lactyltransferase and delactylase and cancer therapy

### HAT families and AARS as lactyltransferases

**Fig. 4:**
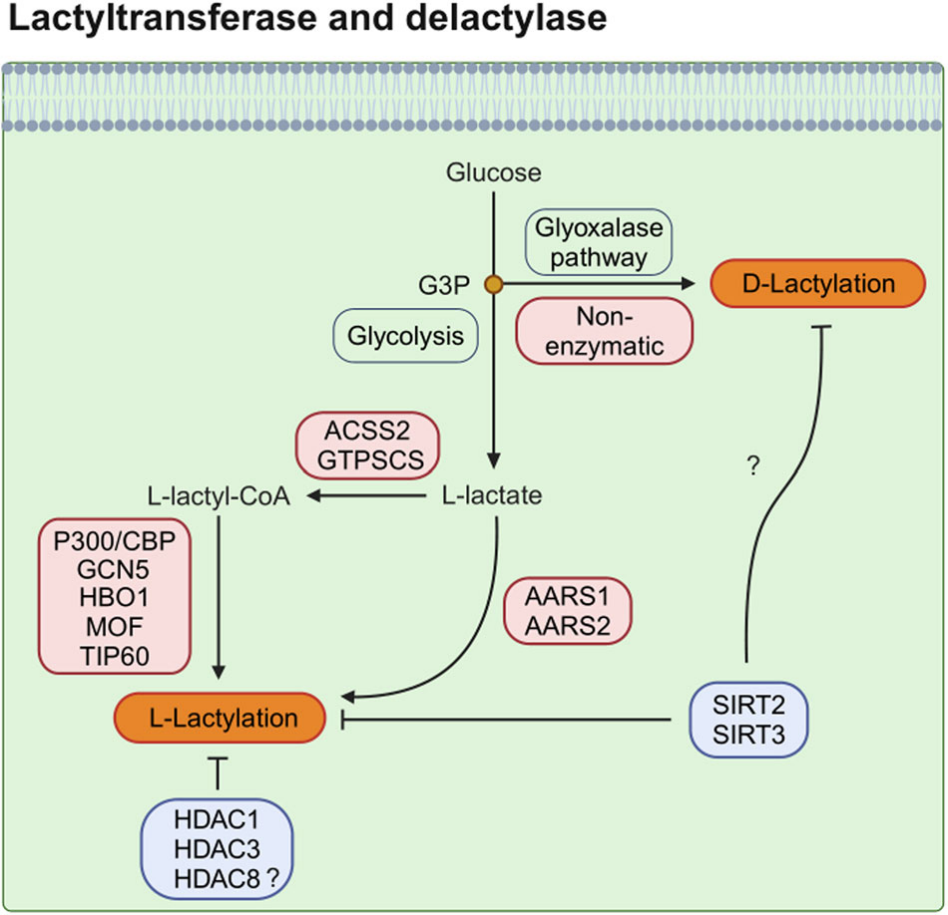
Potential lactyltransferase and delactylase. ACSS2 and GTPSCS activate l-lactate into l-lactyl-CoA, which is subsequently catalyzed by HATs, including p300/CBP, GCN5, HBO1, MOF and TIP60, to install l-lactylation. In addition, l-lactate can be directly activated by AARS to lactate-AMP and then transferred to target proteins for l-lactylation. In contrast to l-lactylation, d-lactylation can be caused by NECMs from metabolites in the GLO pathway, which branches off from G3P in glycolysis. The class I and III (sirtuins) HDAC families are reported as potential delactylases, although further investigations are needed. Enzymes that install and remove lactylation are outlined in red and blue, respectively. SIRT, silent information regulator.

Short-chain lysine acylation is governed by a dynamic balance between the enzymatic activities of HATs and HDACs, which install and remove PTM marks, respectively^11^. As the direct modification of lysine by lactate is considered chemically unfavorable, it was hypothesized that lactate is activated into lactyl-CoA, which may then be transferred by HATs^8,61^. Among the three major HAT families, including the GNAT (Gcn5-related *N*-acetyltransferase), p300/CREB-binding protein (CBP) (p300/CREB-binding protein) and MYST (Moz, Ybf2, Sas2 and Tip60) families, p300/CBP exhibits broad and promiscuous substrate specificity. In a study that initially identified histone Kla, p300 was suggested to be a potential lactyltransferase of histone Kla, although the kinetics of histone lactylation by p300 are much slower than those of histone acetylation^8^. Another study reported that p300 or its counterpart CBP can install Kla on nonhistone proteins (Fig. 4)^13^. In addition to p300/CBP, general control nonderepressible 5 (GCN5), HAT binding to ORC1 (HBO1), males absent on the first (MOF) and Tat-interactive protein 60 (TIP60) in the MYST family are reported as potential lactyltransferases (Fig. 4)^50,55,62,63^.

HATs install Kla from the activated form of lactate, lactyl-CoA. Recently, the enzymes involved in the synthesis of lactyl-CoA in mammalian cells, such as lactyl-CoA synthetase, have been identified. One study suggested that GTPSCS is a lactyl-CoA synthetase that interacts with p300 in the nucleus to install H3K18la^25^. Another study proposed that acetyl-CoA synthetase 2 (ACSS2) functions as a lactyl-CoA synthetase that locally produces lactyl-CoA to provide substrates to GCN5, thus inducing H3K14 and H3K18la^63^.

In addition to HAT-mediated lactyltransferase mechanisms, interesting observations have recently revealed that AARS can be a potential lactyltransferase that directly senses lactate and transfers the lactyl moiety to target proteins (Fig. 4)^16–18^. Owing to the structural similarity between lactate and its native substrate, alanine, AARS1 and AARS2 (cytosolic and mitochondrial isoforms, respectively) may participate in cytosolic and mitochondrial Kla modification, although their main function is the attachment of alanine to its corresponding tRNA^16,64^. In addition, a recent study revealed that cytosolic AARS1 has a conserved nuclear localization signal motif, suggesting the possibility that AARS1 is a potential lactyltransferase in the nucleus in response to lactate^16^. Indeed, AARS1 is elevated in various cancers, and it modifies the Kla of p53, which prevents p53 from binding to DNA, thus impeding the tumor suppressive function of p53 (ref. ^17^). In gastric cancer, AARS1 lactylates YAP, which accelerates cancer cell proliferation^16^. In contrast, another study has shown that AARS1 and AARS2 regulate Kla on METTL16, which causes copper-induced gastric cancer cell death^65^. In macrophages, AARS2 lactylates cGAS, which reduces the affinity of cGAS for DNA, thus resulting in poor immune surveillance^18^.

### Class I HDACs and SIRTs as delactylases

HDACs have four classes depending on domain structure and cofactors, including Zn^2+^-dependent classes I, II and IV and NAD^+^-dependent class III HDACs^66^. Class I HDACs, including HDAC1, 2, 3 and 8, are primarily localized in the nucleus^67^. Class II HDACs can be divided into two subgroups, namely, IIa (including HDAC4, 5, 7 and 9) and IIb (including HDAC6 and 10). Class IIa HDACs translocate between the cytosol and nucleus in response to signal-dependent phosphorylation, whereas class IIb HDACs are primarily localized to the cytosol^68^. Class III HDACs include seven NAD^+^-dependent Sirtuins (SIRT1–7) that are homologous to yeast Sir2, and their deacetylase activity on histones and nonhistone proteins depends on their subcellular localization^69^. Finally, class IV HDACs include only HDAC11^70^.

Currently, class I HDACs and class III HDACs (sirtuins) have been shown to have delactylase activity, but this remains controversial (Fig. 4). In 2021, SIRT2 emerged as a potential delactylase^71^. Interestingly, the study induced Kla through the GLO pathway, resulting in nonenzymatic Kla modification (further discussed in the following section). The authors suggest that a nonenzymatic reaction generates d-lactyllysine, whereas l-lactyl-CoA from the conversion of pyruvate into l-lactate generates l-lactyllysine. They concluded that SIRT2 has a propensity to remove l-lactyllysine but has minimal activity on d-lactyllysine^71^. Hence, further investigation is needed to clarify whether SIRT2 plays a role as a delactylase in their nonenzymatic d-lactyllysine modification. Another study demonstrated that SIRT2 is an efficient delactylase of nonhistone proteins, including METTL16 (ref. ^65^). In addition to SIRT2 being a delactylase, SIRT3 was shown to delactylate CCNE2 in HCC, preventing HCC outgrowth^72^. Although SIRT3 is found primarily in mitochondria^69^, SIRT3 is also known to exhibit superior delactylation activity toward H4K16la compared with other human Sirtuin families^73^.

In contrast, Zhao and colleagues, who discovered the first histone Kla, reported that HDAC1–3 and SIRT1–3 act as delactylases in vitro, but HDAC1 and HDAC3 remove Kla from histones in vivo because the pan-sirtuin inhibitor nicotinamide (NAM) does not influence l-lactyllysine on histones in cells^74^. Another study demonstrated that HDAC3 has potent delactylase activity that is several thousand-fold greater than that of SIRT2 (ref. ^75^); thus, HDAC3 may be a delactylase in vivo. However, overexpressed HDAC3 did not significantly decrease H3K18la and pan-Kla modification^74^, as well as nonhistone protein dehydrogenase/reductase 7 (DHRS7) at K321 (ref. ^76^). These findings indicate that HDAC3 possesses site-specific delactylase activities in cells. In addition to HDAC3, HDAC1, which forms multiprotein complexes including MiDAC and RERE, has delactylase activities on histone H2B in the nucleosome context, albeit at relatively moderate rates^77^. Considering that HDAC3 is found primarily in the nucleus and that SIRT2 is found in the cytosol, HDAC3 and SIRT2 may account for the delactylase activities of histones in the nucleus and nonhistone proteins in the cytosol, respectively, although further investigation, including the potential delactylase activity of SIRT2 in the nucleus, is needed because SIRT2 is also found in the nucleus during mitosis^78^.

### Targeting Kla-modifying enzymes as anticancer therapy

Given that histone and nonhistone Kla modifications are reversible and strongly related to poor prognosis in cancers and tumor development, targeting Kla-modifying enzymes may provide a promising strategy for cancer therapy. As there are no specific Kla-modifying enzymes, common epigenetic drugs that target HATs and HDACs are considered potential therapeutic agents. Targeting p300/CBP primarily focuses on inhibiting the HAT domain and bromodomain (BRD)^79^. Among the compounds that target the HAT domain, C646 treatment has been shown to reduce HMGB1 Kla and H3K18la levels^13,32,37^. Another HAT domain-targeting compound, A485, also decreases H3K18la and nonhistone Kla in CRC and HCC, respectively^15,42^. The compounds targeting the BRD domain of p300, including CCS1477 and FT-7051, are currently in clinical trials^80^, and it remains to be determined whether their anticancer effect is mediated by the inhibition of Kla.

As previously indicated, AARS moonlights as a lactyltransferase. Owing to the structural similarity of lactate, β-alanine competes with lactate for binding to AARS and inhibits lactylation globally^17^. Interestingly, β-alanine treatment substantially inhibits the proliferation of cancer cells, while AARS1 depletion does not have adverse effects on impeding protein synthesis^16,17^. Considering that β-alanine supplementation has already been used as an ergogenic aid^81^, Kla regulation by β-alanine may enable a more rational approach to improve clinical outcomes with a manageable toxicity profile. Several HDAC inhibitors (HDACis), including vorinostat, belinostat, panobinostat and romidepsin, have been approved for clinical practice^82^, suggesting that these HDACis also have low toxicity. However, the effects of these HDACis on histone and nonhistone Kla need to be thoroughly investigated to develop novel anticancer therapeutic strategies because inhibiting HDACs may increase Kla, resulting in an unexpectedly poor prognosis in cancer treatment.

## Nonenzymatic lactylation by LGSH and glyceraldehyde-3-P

**Fig. 5:**
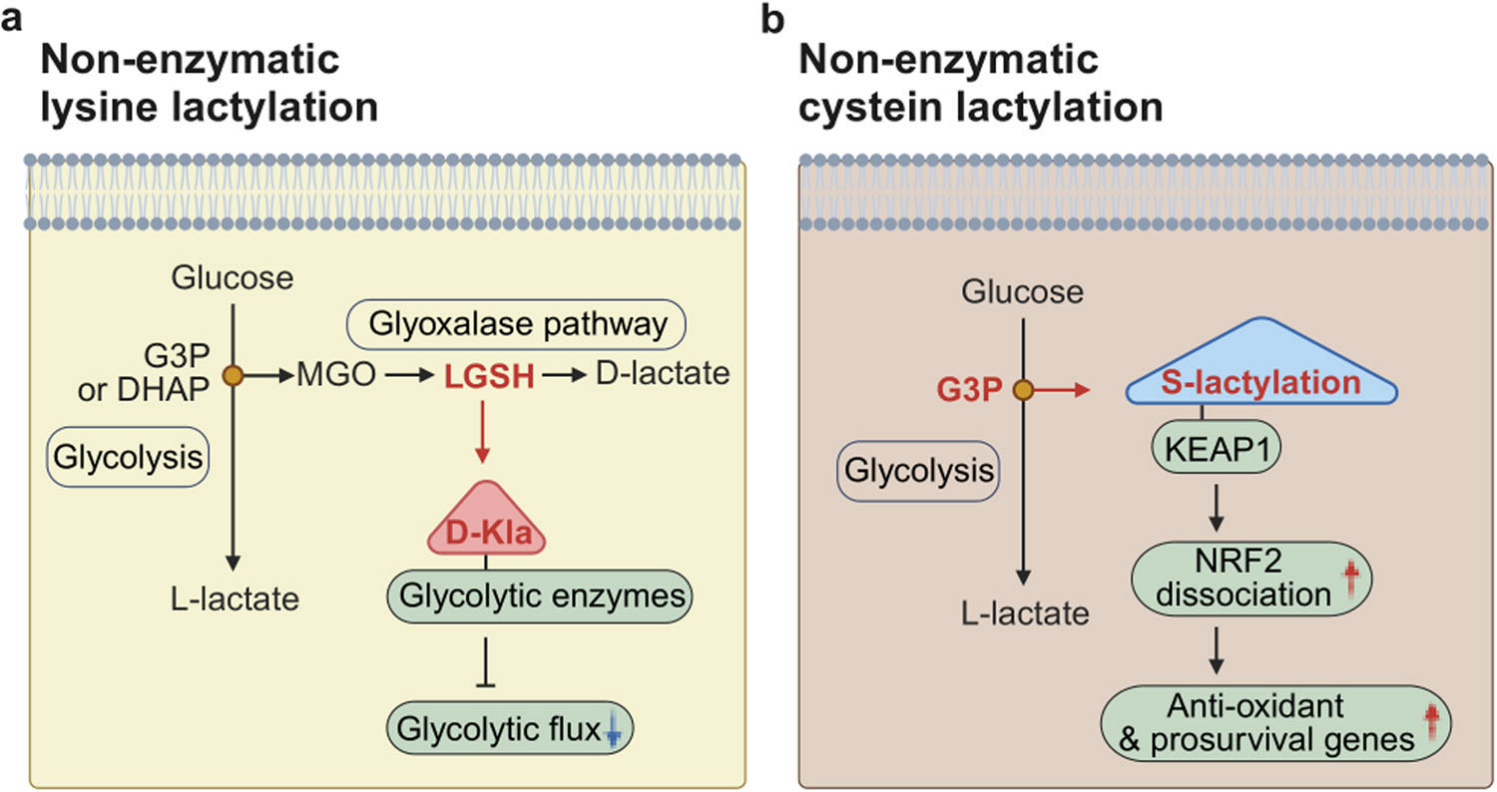
Nonenzymatic lactylation at lysine and cysteine residues. **a** LGSH from the GLO pathway modifies protein lysine residues nonenzymatically to produce d-lactyllysine. This modification primarily occurs in glycolytic enzymes and inhibits their activity to form negative feedback. **b** G3P from glycolysis modifies cysteine residues (*s*-lactylation) of KEAP1, which liberates NRF2. Dissociated NRF2 translocates into the nucleus and induces NRF2-dependent gene expression. D-Kla, d-lysine lactylation.

PTMs are commonly introduced onto proteins by an enzyme-mediated process, but reactive glycolytic metabolites such as 1,3-bisphosphoglycerate (1,3BPG) are known to modify target proteins by NECM, such as 3-phosphoglyceryl-lysine modification^83^. Kla modification can also occur nonenzymatically through reactive metabolites formed from the GLO pathway, which branches off from the glycolysis intermediates DHAP and G3P (Fig. 5a)^21^. These triose phosphate metabolites spontaneously produce methylglyoxal (MGO) via the nonenzymatic elimination of phosphate. MGO is a highly reactive dicarbonyl compound, so two consecutive enzymatic reactions catalyzed by glyoxalase 1 (Glo1) and glyoxalase 2 (Glo2) detoxify it to d-lactate using glutathione (Fig. 1)^20^. Glo1 expression in cancer cells is elevated to prevent increased intracellular MGO concentrations, leading to LGSH production^84,85^. LGSH is then hydrolyzed by Glo2 to restore glutathione by releasing d-lactate (Fig. 1). Interestingly, MGO is known to primarily modify arginine in histones to form hydroimidazolone derivatives (MG-H), especially in Glo1 knockout cells^86^, but LGSH modifies lysine nonenzymatically to d-lactyllysine in Glo2 knockout cells^21^. Furthermore, Galligan and colleagues reported that d-lactyllysine modification primarily occurs in glycolytic enzymes, inhibiting their activity to form a feedback mechanism (Fig. 5a)^21^. Recently, an interesting observation revealed that a small molecule inhibitor of PKM2, sAKZ692, accumulates G3P, which results in elevated lactylation, notably at the cysteine residues (s-lactylation) of Kelch-like ECH-associated protein (KEAP1)^22^, the repressor protein of nuclear factor erythroid 2-related factor 2 (NRF2). s-lactylation of KEAP1 subsequently dissociates NRF2 and induces NRF2-dependent transcription of antioxidant genes (Fig. 5b). Given that the accumulation of G3P leads to the production of reactive metabolites, such as MGO, which increases reactive oxygen species levels, cancer cells may develop defense mechanisms against reactive oxygen species by s-lactylation.

As many cancer cells upregulate Glo1 expression to protect against MGO-induced cytotoxicity, Glo1 inhibition may represent an anticancer therapeutic strategy. *S*-*p*-Bromobenzylglutathione cyclopentyl diester is a Glo1 inhibitor that induces MGO accumulation and inhibits tumor growth^87^. Methotrexate, a chemotherapeutic drug approved by the Food and Drug Administration, inhibits Glo1 in patients with acute lymphoid leukemia^88^. Considering that Glo1 inhibition diminishes LGSH production, whether these drugs display an anticancer effect by inhibiting LGSH-induced d-lactylation remains to be determined.

## Conclusions and future perspectives

Reprogrammed glucose metabolism in cancer cells has been a subject of therapeutic interest^3^. However, cancer cells often exhibit metabolic flexibility to overcome therapeutic interventions, making this approach challenging to pursue in clinical practice. Since most cancer cells are highly glycolytic, it is reasonable to assume that histone and nonhistone proteins modifications by lactylation are common phenomena in cancer cells. Thus, reversing lactylation could lead to a promising anticancer therapeutic strategy. To exploit the inhibition of lactylation as an anticancer therapy, several critical challenges remain to be addressed. For example, potential Kla-modifying enzymes exert diverse types of short-chain lysine acylation, so achieving selectivity for Kla-specific modulation is needed. Another important concern is the existence of stereoisomeric Kla, such as l- and d-lactylation^89^, although the most recent study revealed that l-lactylation is the major Kla regulated by glycolysis^24^. As Kla-modifying enzymes, including HDAC3 and SIRT2, have distinct preferences for d- and l-lactylation, respectively^71,74^, studying a major lactylation isomer and its functional characterization as well as different half-lives may lead to new and efficacious anticancer therapeutic strategies. It is also important to establish which is the predominant enzyme that mediates lactylation and whether different enzymes mediate lactylation at different cellular localizations. Furthermore, it is necessary to study the potential crosstalk between lactylation and other PTMs to deepen our understanding. In summary, increasing evidence suggests that lactylation plays a crucial role in cancer development, and its inhibition shows promising anticancer effect at the preclinical level. To achieve the goal of curing cancer in patients by targeting lactylation, further investigations are needed to successfully translate current advances to clinical trials.
